# A Non-Linear Relationship between Preoperative Total Bilirubin Level and Postoperative Delirium Incidence after Liver Transplantation

**DOI:** 10.3390/jpm12020141

**Published:** 2022-01-21

**Authors:** Ru-Yi Lu, Heng-Kai Zhu, Xiang-Yan Liu, Li Zhuang, Zhuo-Yi Wang, Yuan-Li Lei, Ting Wang, Shu-Sen Zheng

**Affiliations:** 1 Department of Neurology, Shulan (Hangzhou) Hospital, Shulan International Medical College, Zhejiang Shuren University, Hangzhou 310022, China; ruyi.lu@shulan.com; 2 Department of Hepatobiliary and Pancreatic Surgery, Shulan (Hangzhou) Hospital, Shulan International Medical College, Zhejiang Shuren University, Hangzhou 310022, China; zhuhengkai@zju.edu.cn (H.-K.Z.); xiangyan.liu@shulan.com (X.-Y.L.); li.zhuang@shulan.com (L.Z.); zhuoyi.wang@shulan.com (Z.-Y.W.); ting.wang@shulan.com (T.W.); 3 Department of Hospital Medicine, Marshfield Medical Center, 1000 North Oak Avenue, Marshfield, WI 54449, USA; yuanlilei91@gmail.com

**Keywords:** total bilirubin, postoperative delirium, liver transplantation, saturation effect, generalized additive models

## Abstract

This study aimed to explore the correlation between preoperative total bilirubin (TBil) level and postoperative delirium (POD) in orthotopic liver transplantation (OLT). All the OLT consecutively performed between April 2019 and March 2021 were retrospectively reviewed with data retrieved from a prospectively collected database. Logistic regression model and generalized additive model were used to identify both linear and non-linear relationships between TBil and POD. A two-piecewise regression model was performed to calculate the saturation effect. Subgroup analyses were performed using stratified logistic regression models. A total of 402 recipients were enrolled. After fully adjusted for covariates, TBil was indicated to have a non-linear relationship with POD. The two-piecewise regression model showed the inflection point was 20 mg/dL. On the left side of the inflection point, the incidence of POD increased by 5% per 1 mg/dL increment of TBil (*p* = 0.026). On the right side of the inflection point, the effect size had no statistical significance (OR, 0.97; 95% CI, 0.90–1.05; *p* = 0.482). The relationship between preoperative TBil level and POD incidence is non-linear in OLT recipients. The incidence of POD is positively correlated with TBil level when it is below 20 mg/dL. A saturation effect is observed when TBil level reaches 20 mg/dL.

## 1. Introduction

In recent years, orthotopic liver transplantation (OLT) has been widely accepted as an imperative treatment option for all forms of end-stage liver diseases. Neurological complications remain one of the major challenges after OLT, with a reported incidence of up to 30% [1]. As a common neuropsychiatric complication, postoperative delirium (POD) is an acute state characterized by impairment of consciousness, perception, attention, or orientation with a fluctuating pattern [2]. Studies have pointed out that the pooled incidence of POD after OLT is approximately 30% [3].

Recent studies have focused on the effects of POD on short-term and long-term outcomes after operation [3,4,5,6,7,8,9,10,11]. Delirium is demonstrated to extend hospital stay, increase hospitalization costs, as well as increase the risk of mortality, morbidity, and cognitive impairment after surgery, as illustrated among other populations other than OLT [2,12,13,14]. Zhou et.al showed that the occurrence of POD after OLT was associated with prolonged mechanical ventilation, hospital length of stay and increased renal replacement therapy (RRT) in intensive care unit (ICU) [3]. Additionally, patients with POD were at a three- to fourfold increased risk of the hospital mortality and one-year mortality [3]. Similarly, OLT recipients with POD were more likely to have longer hospital stays (27.6 vs. 11.2 days, *p* = 0.003) and higher 6-month mortality (13.2% vs. 1.4%, *p* = 0.003) than patients who did not develop delirium in Oliver’s study [8]. Moreover, patients with POD had a trend toward increased frequency of hospital acquired infections, including urinary tract infections and pneumonia [10]. Furthermore, POD was associated with a long-term cognitive decline [15]. In a prospective cohort study conducted by Austin et.al, patients with POD appeared to attenuate the improvement in cognition 90 days after surgery [7]. However, the pathophysiological mechanisms of delirium are poorly understood, and there is no single intervention or medication to treat delirium [16]. Therefore, early identification of risk factors, and consequently prompting treatment with multimodal strategies is a critical and urgent challenge.

Several risk factors have been identified to facilitate POD development, including the model for end-stage liver disease (MELD) scores, APACHE II scores, presence of hepatic encephalopathy, preoperative ammonia level, and preoperative TBil level [3,17]. However, some studies summarize contrary findings [18]. Whether bilirubin correlates with the incidence of POD is controversial, and both the mechanism of bilirubin-induced neurotoxicity and delirium are indeterminate. The modulation of neurotransmitter metabolism, interactions with biological membranes, regulation of enzyme activities and inflammatory cytokine were suggested as major determinants of bilirubin-induced neurotoxicity [19], which may be involved in the pathophysiological mechanisms of delirium.

However, studies evaluating the effects of TBil on POD incidence are limited due to small sample size. Park et.al concluded that preoperative bilirubin above 3.5 mg/dL was associated with increased risk of POD, though the cutoff value was rather lower than average in transplanted recipients [17]. Thus, this retrospective cohort study aimed to investigate the correlation between preoperative TBil and POD with a larger sample size, as well as more informative statistical methods.

## 2. Patients and Methods

### 2.1. Patient Selection and Data Collection

All the OLT consecutively performed at Shulan (Hangzhou) Hospital between 1 April 2019, and 31 March 2021, were retrospectively reviewed. Exclusion criteria were defined as: (1) patients under 18 years old; (2) patients underwent re-transplantation; (3) patients with missing data or unevaluable mental status. All the data including demographic, pre-, peri-, and post-transplant parameters of donors and recipients were retrieved from a prospectively collected database. We confirmed all organs were from voluntary donation with written informed consent. We guaranteed that no organs of prisoners, coerced, or paid individuals used in this study. This study was reviewed and approved by the Ethics Committee of Shulan (Hangzhou) Hospital. It was conducted in accordance with the Declaration of Istanbul and the Declaration of Helsinki.

### 2.2. Perioperative Protocol

General anesthesia was induced using 2–4 µg/kg of fentanyl and a muscle relaxant such as 0.6–1 mg/kg of rocuronium or 0.1–0.2 mg/kg of cisatracurium. Inhaled sevoflurane was administered in an oxygen/gas mixture with 50–60% oxygen. Remifentanil (0.5–10 µg/kg/h) and a muscle relaxant (0.3–0.6 mg/kg/h of rocuronium or 0.1–0.2 mg/kg/h of cisatracurium) were infused during the operation.

A modified piggyback transplant procedure was performed. Following the surgery, the patients were transferred to the ICU. When patients regained consciousness showing spontaneous breathing efforts with stable vital signs, extubation was performed. The immunosuppressant regimen consisted of 2 doses of basiliximab on days 0 and 4, intraoperative 1000 mg methylprednisolone, postoperative tacrolimus, and 720 mg/day mycophenolate sodium. The tacrolimus starts with 0.1 mg/kg/day on day 3 and target trough level was 8–10 ng/mL during the first month after OLT.

### 2.3. Postoperative Mental Status Assessment

The postoperative mental status of recipients in the ICU was routinely assessed by trained clinical practitioners twice a day and whenever patients developed a mental change. POD was evaluated using the Diagnostic and Statistical Manual of Mental Disorders-IV (DSM-IV) criteria or the Confusion Assessment Method for the ICU (CAM-ICU) scale. The assessments commenced on the first day after the patient’s operation and continued for 7 days, or until either discharge from the ICU or the death of the patient.

### 2.4. Diagnosis of POD

POD was defined as an acute-onset neuropsychiatry state with fluctuating course of confusion or alteration of mental status within 7 days after OLT. The diagnosis was confirmed if (1) patients had symptoms and signs consistent with the DSM-IV criteria for delirium and were treated with haloperidol or other medications, or (2) they were diagnosed by using the CAM-ICU scale with psychiatry or neurological consultation.

### 2.5. Statistical Analysis

To group TBil in tertiles, the data of TBil were ordered from smallest to largest and the ordered distribution was divided into three parts of more-or-less equal size. Patients were classified into three groups by tertiles of TBil levels and the baseline characteristics were compared among the groups. Continuous variables were expressed as the means ± standard deviations (normal distribution) or medians (interquartile range) (skewed distribution), and categorical variables were expressed as a frequency or percentages. The one-way ANOVA (normal distribution), Kruskal–Wallis H (skewed distribution) test, and chi-square test (categorical variables) were used to determine any significant differences among the groups. Secondly, univariate analysis with logistic regression was performed to evaluate the association between the variables and POD. Then, the results of the crude, minimally adjusted, and fully adjusted models were simultaneously shown, which focused on the relationship between TBil and POD. We examined the relationship of TBil as a continuous variable and as categorized into tertiles with the risk of POD. The covariates changing the matched odds ratio (OR) by at least 10% when added to the model or the covariates with *p* < 0.1 in the univariate model would be adjusted. The results were expressed by the effect size with OR and 95% confidence interval (CI).

Furthermore, generalized additive models (GAM) were used to identify non-linear relationships between TBil and POD. Then a two-piecewise regression model was performed to calculate the saturation effect of TBil on POD in terms of the smoothing spline. Lastly, subgroup analyses were performed using stratified logistic regression models. The modifications and interactions of subgroups were inspected by likelihood ration tests. All of the analyses were performed with the statistical software package R (http://www.R-project.org, 12 January 2022, The R Foundation) and Empowerstats (http://www.empowerstats.com, 12 January 2022, X&Y Solutions, Inc., Boston, MA, USA). *p* values less than 0.05 were considered statistically significant.

## 3. Results

### 3.1. The Selection of Enrolled Patients

**Figure 1:**
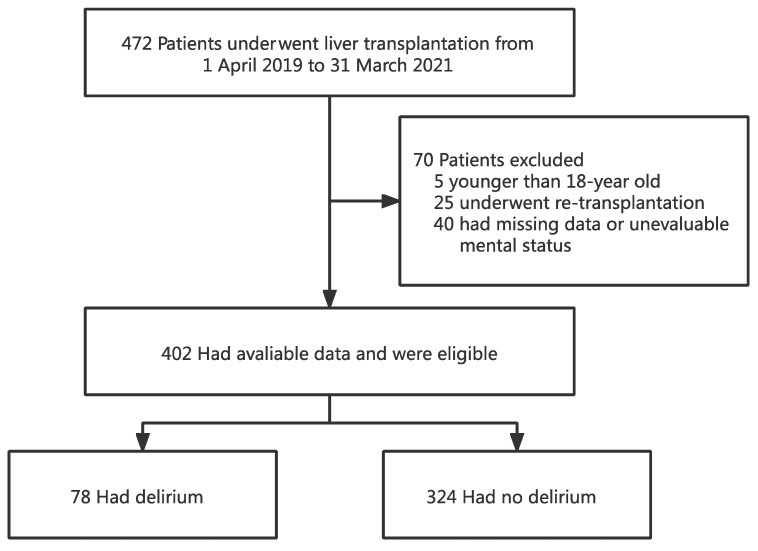
Flowchart analyzing the effect of preoperative total bilirubin on postoperative delirium after liver transplantation.

A total of 472 patients underwent OLT between 1 April 2019 and 31 March 2021. Of the 472 recipients, 402 were eligible for the study and 70 were excluded. Of the 70 excluded subjects, 5 were younger than 18 years old, 25 underwent re-transplantation, and the remaining 40 had missing data or were unable to assess the mental status due to coma or deep sedation (Figure 1). 

### 3.2. Baseline Characteristics of the Patients

Baseline characteristics are listed in Table 1. There were no statistically significant differences in gender, creatinine, hepatitis C, alcohol consumption, diabetes mellitus, hypertension, cardiovascular disease, donation after brain death (DBD), graft weight, auto-transfusion, post-transplant ammonia, or post-transplant sedatives among different TBil groups.

The highest TBil group (T3) had a younger age, higher MELD score, higher international normalized ratio (INR), higher TBil level, and lower serum sodium (*p* < 0.05). Compared with the group T3, significantly less patients were transplanted for malignancy, transplanted with encephalopathy, or underwent preoperative artificial liver support and RRT in other two groups (T1 and T2). Group T3 had higher intraoperative bleeding and was transfused with more red blood cell (RBC) and fresh frozen plasma (FFP) (*p* < 0.05). The intraoperative urine output was lower and more RRT was required in Group T3. The risk of POD was about three times higher in the high bilirubin level group (T3) than the low bilirubin group (T1) (29.8% vs. 10.1%, *p* < 0.001).

### 3.3. Univariate Analysis

The univariate analysis showed that the TBil level, INR, malignancy, encephalopathy, artificial liver support, intraoperative auto-transfusion, and post-transplant ammonia were positively correlated with the incidence of POD (Table 2). While the other factors, including age, gender, body mass index (BMI), creatinine, serum sodium, alcohol consumption, diabetes mellitus, hypertension, cardiovascular disease, hepatitis B, RRT, DBD, ABO compatibility, intraoperative transfusion and bleeding, and intraoperative RRT were found to have no significant correlations with POD (*p >* 0.05).

### 3.4. The Relationship between TBil and POD

Three models were used to evaluate the linear association between TBil and POD (Table 3). In the crude model, the incidence of POD increased 4% with each 1 mg/dL increment of TBil (OR, 1.04; 95% CI, 1.02–1.07; *p* < 0.001). In the minimally adjusted model for age and gender, the association was also identified (OR, 1.05; 95% CI, 1.02–1.07; *p* < 0.001). However, in the fully adjusted model, we did not conclude the linear relationship between TBil and POD (*p* > 0.05). For sensitivity analysis, the TBil was handled as a categorical variable (tertiles) and a significant trend of POD risk increasing was observed (all *p* < 0.05 in three models).

### 3.5. The Analyses of Non-Linear Relationship

**Figure 2:**
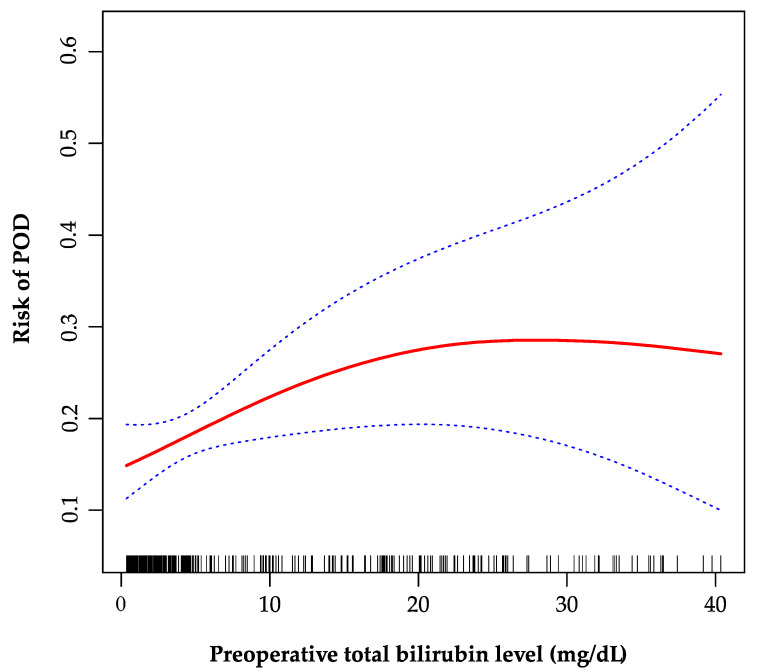
The non-linear relationship between preoperative total bilirubin and POD. A non-linear relationship between them was identified after adjusting for age, gender, creatinine, international normalized ratio, sodium, malignancy, encephalopathy, artificial liver support, auto-transfusion, intraoperative renal replacement therapy, and post-transplant ammonia. POD, postoperative delirium.

As is shown in the smoothing spline, the TBil was indicated to have a non-linear relationship with POD (Figure 2). The two-piecewise regression model showed the inflection point was 20 mg/dL after fully adjusted for covariates (age, gender, creatinine, INR, sodium, malignancy, encephalopathy, artificial liver support, auto-transfusion, intraoperative RRT, and post-transplant ammonia). On the left side of the inflection point, the incidence increased 5% per 1 mg/dL in TBil, with 95% CI between 1.01 and 1.11, *p* = 0.026 (Table 4). On the right side of the inflection point, the effect size had no statistical significance (OR, 0.97; 95% CI, 0.90–1.05; *p* = 0.482).

### 3.6. The Results of Subgroup Analyses

As shown in Table 5, the tests for interactions were significant for BMI and artificial liver support. The effect sizes of TBil on POD showed significant differences in different body sizes (*p* for interaction = 0.006). TBil was positively associated with POD in recipients whose BMI ≤ 22 (OR, 1.07; 95% CI, 1.01–1.14). Additionally, the adjusted OR (95% CI) for POD in recipients did not receive artificial liver support was 1.03 (1.00, 1.07) compared with 0.99 (0.93, 1.05) those received (*p* for interaction = 0.036).

We did not find any statistically significant interactions in age, gender, MELD, malignancy, encephalopathy, alcohol consumption, diabetes mellitus, hypertension, intraoperative bleeding and transfusion, intraoperative urine output and RRT, or post-transplant ammonia and sedatives.

## 4. Discussion

In this study, generalized linear model (GLM), id est, logistic regression, and GAM were used to elaborate the relationship between preoperative TBil level and POD. The fully adjusted GLM showed that there was no statistical linear relationship between TBil and POD. When the tertiles of TBil was regarded as categorical variable, the significant trend was observed. Furthermore, the GAM model and two-piecewise logistic regression model indicated that the relationship between TBil and POD was non-linear. To the best of our knowledge, this non-linear relationship has not been reported elsewhere. The correlations were different on the left and right sides of the inflection point (TBil = 20 mg/dL). On the left side of the inflection point, the incidence of POD increased by 5% per 1 mg/dL in preoperative TBil level, while on the right side the effect appeared to be saturated.

POD is a frequent and serious complication after OLT as it is closely associated with increased morbidity, mortality, and medical care expenses [8,20,21,22]. It was demonstrated to increase hospital stay by 2–3 days and was associated with a 30-day mortality of 7–10% in recent literature [5]. Most OLT recipients are at an extremely complex condition with nutritional, metabolic, and electrolyte imbalances. Besides the operation procedure, extensive intraoperative blood loss and transfusion can also worsen the condition, exposing the recipients to higher risk of POD. A recent meta-analysis reported the pooled incidence of POD in OLT was 30% [3], ranging from 10% to 47% [20,21]. The result was comparable to our estimates (19.4%, with 78 in 402 patients). More efforts looking for novel and unique risk factors of POD after OLT are urgently needed.

Many reports have focused on a variety of predictive factors, lessoned from which the univariate analysis in our study was conducted [3,6,17,18]. Though the disorder was multifactorial, the role of TBil has caused much concern among these factors. Several studies found that the differences in preoperative TBil between POD and non-POD group had no statistical significance [11,20,21]. Yet, Yoon et.al showed patients with POD tended to have a higher level of bilirubin but no further investigation was continued [23]. Park’s study indicated that preoperative TBil level above 3.5 mg/dL was significantly associated with POD [17]. However, the conclusion seemed debatable since the preoperative TBil level was usually higher in transplant recipients, indicating the severity of the primary disease. The cutoff value as 3.5 mg/dL appeared to be of less value for clinical practice. Taken together, the results of these studies were conflicting. Our study demonstrated the positive correlation with saturation effect between TBil and POD, which fits more to our clinical observation.

As a crucial component of MELD score, the level of TBil is a surrogate marker of the severity of primary liver diseases. In the presence of severe liver disease, excess unbound bilirubin entered the blood–brain barrier [24,25]. The neurotoxic effects of the bilirubin have been described in literature, especially in newborns [25]. Hansen et.al summarized a complex range of mechanisms for bilirubin neurotoxicity, including inhibition of cell respiration, interaction with membrane components, regulation of neurotransmitter and enzyme activity, induction of apoptosis of neurons, and involvement of infection and immunology [19]. Consistently, similar mechanism of POD involving neuroinflammation and neurotransmitter regulation were supported by animal models [5]. Hence, we speculated the high level of TBil induced neurotoxic effects in OLT recipients through similar pathways, resulting in the confusion of awareness as delirium. The saturation effect may arise from the hypothesis that inhibition of key molecular in neurotransmission, such as acetylcholinesterase, no longer increases the effect on acetylcholine after bilirubin level exceeds the inflection point. This allows acetylcholine to be retained in the synaptic cleft and blocks a new stimulus transmission. The exact mechanism needs more studies to be uncovered in the future.

This study has its strengths. It utilizes both GLM and GAM to clarify the linear and non-linear relationships between TBil and POD. The use of GAM fits a smoothing spline and helps us better understand the real relationship between the variable and the outcome, and rigorous statistical adjustment is performed for confounding factors, due to the retrospective nature. The interaction analysis improves the reliability of the data. Notably, the study concludes the positive correlation that when the TBil level is lower than 20 mg/dL, the incidence of POD increases by 5% for every 1 mg/dL increase in TBil. Such correlation can be used in clinical practice as a predict tool assessing the risk of POD. Moreover, the saturation effect of TBil level above 20 mg/dL indicates the peak severe influence on postoperative neuropsychiatric events.

There are some limitations of our study. This is a retrospective cohort study, providing weaker evidence about the association between the exposure and the outcomes than prospective studies. Accurate diagnosis of POD faces many challenges due to the fluctuating pattern of the symptoms. Patients’ baseline mental status can also be difficult to be evaluated due to existing encephalopathy or perioperative sedations. Moreover, patients with high preoperative TBil level are more vulnerable to neuropsychiatric complications before OLT and consequently, are more likely to be ruled out because of unevaluable mental status. Therefore, the incidence of POD may be underestimated. Thus, more prospective studies with larger sample sizes are required to validate the relationship between the risk factors and POD.

## 5. Conclusions

The relationship between the preoperative TBil and POD is non-linear in OLT recipients. The incidence of POD is positively correlated with TBil when the level of TBil is below 20 mg/dL. A saturation effect is observed when TBil level reaches 20 mg/dL.

## Figures and Tables

**Table 1:** Baseline characteristics of patients underwent liver transplantation.

Preoperative Total Bilirubin Level	T1	T2	T3	*p* Value
*n*	139	139	124	
Pre-transplant characteristics				
Age, years, mean ± SD	54 ± 10	51 ± 10	50 ± 10	0.003
Male, *n* (%)	122 (87.8%)	116 (83.5%)	101 (81.5%)	0.349
BMI, mean ± SD	22.6 ± 2.9	24.0 ± 4.0	22.8 ± 3.7	0.002
MELD score at transplant, median (IQR)	10 (8–12)	16 (14–20)	28 (24–33)	<0.001
Total bilirubin, mg/dL, mean ± SD	1.1 ± 0.4	4.3 ± 2.4	22.1 ± 7.6	<0.001
Creatinine, mg/dL, mean ± SD	0.9 ± 0.6	0.8 ± 0.3	1.0 ± 0.9	0.076
INR, mean ± SD	1.3 ± 0.3	1.7 ± 0.5	2.4 ± 1.0	<0.001
Serum sodium, mmol/L, median (IQR)	140 (138–141)	138 (135–141)	136 (133–139)	<0.001
Liver diseases				
Hepatitis B, *n* (%)	118 (84.9%)	103 (74.1%)	88 (71.0%)	0.018
Malignancy, *n* (%)	37 (26.6%)	57 (41.0%)	73 (58.9%)	<0.001
Hepatitis C, *n* (%)	1 (0.7%)	1 (0.7%)	2 (1.6%)	0.692
Encephalopathy, *n* (%)	6 (4.3%)	19 (13.7%)	45 (36.3%)	<0.001
Artificial liver support, *n* (%)	1 (0.7%)	13 (9.4%)	57 (46.0%)	<0.001
RRT, *n* (%)	1 (0.7%)	6 (4.3%)	13 (10.5%)	0.001
Alcohol consumption, *n* (%)	22 (15.8%)	30 (21.6%)	26 (21.0%)	0.416
Diabetes mellitus, *n* (%)	26 (18.7%)	18 (12.9%)	17 (13.7%)	0.352
Hypertension, *n* (%)	31 (22.3%)	28 (20.1%)	19 (15.3%)	0.347
Cardiovascular disease, *n* (%)	9 (6.5%)	7 (5.0%)	5 (4.0%)	0.669
Donation and transplantation surgery				
DBD, *n* (%)	57 (41.0%)	65 (46.8%)	48 (38.7%)	0.390
Graft weight, g, mean ± SD	1447 ± 352	1488 ± 349	1486 ± 388	0.575
ABO compatible transplantation, *n* (%)	132 (95.0%)	127 (91.4%)	97 (78.2%)	<0.001
Intraoperative transfusion				
RBC, u, median (IQR)	2.0 (0.0–6.0)	4.0 (2.0–8.5)	6.0 (4.0–9.0)	<0.001
FFP, mL, median (IQR)	750 (600–970)	800.0 (650–1085)	845 (750–1170)	<0.001
Auto-transfusion, mL, median (IQR)	250 (0–500)	250 (0–500)	250 (0–500)	0.444
Intraoperative bleeding, mL, median (IQR)	1000 (800–1500)	1500 (1000–2000)	1500 (1000–1850)	<0.001
Intraoperative urine output, mL, median (IQR)	1200 (800–1675)	1050 (700–1600)	810 (300–1350)	<0.001
Intraoperative RRT, *n* (%)	6 (4.3%)	8 (5.8%)	29 (23.4%)	<0.001
Post-transplant ammonia, umol/L, median (IQR)	50 (39–66)	46 (34–64)	49 (32–75)	0.298
Post-transplant sedatives, *n* (%)	29 (20.9%)	26 (18.7%)	25 (20.2%)	0.900
Postoperative delirium, *n* (%)	14 (10.1%)	27 (19.4%)	37 (29.8%)	<0.001
Total bilirubin in day 7 after LT, mg/dL, median (IQR)	1.1 (0.8–1.7)	1.7 (1.3–2.7)	3.3 (2.3–5.7)	<0.001

*p* < 0.05 was considered statistically significant. BMI, body mass index; DBD, donation after brain death; FFP, fresh frozen plasma; INR, international normalized ratio; IQR, interquartile range; LT, liver transplantation; MELD, model for end-stage liver disease; RBC, red blood cell; RRT, renal replacement therapy.

**Table 2:** Predictors of post-transplant delirium by univariate analysis.

	Statistics	OR (95%CI)	*p* Value
Age	52 ± 10	1.01 (0.98, 1.03)	0.578
Gender			
Male	339 (84.3%)	1	
Female	63 (15.7%)	0.86 (0.43, 1.74)	0.671
BMI	23.1 ± 3.6	1.03 (0.96, 1.10)	0.375
Total bilirubin	8.7 ± 10.1	1.04 (1.02, 1.07)	<0.001
Creatinine	0.9 ± 0.6	1.36 (0.98, 1.90)	0.070
INR	1.8 ± 0.8	1.60 (1.23, 2.14)	<0.001
Sodium	138 (135–141)	0.98 (0.96, 1.00)	0.095
Hepatitis B			
No	93 (23.1%)	1	
Yes	309 (76.9%)	0.84 (0.48, 1.49)	0.559
Malignancy			
No	235 (58.5%)	1	
Yes	167 (41.5%)	1.86 (1.13, 3.06)	0.015
Encephalopathy			
No	332 (82.6%)	1	
Yes	70 (17.4%)	2.92 (1.65, 5.17)	<0.001
Artificial liver support			
No	331 (82.3%)	1	
Yes	71 (17.7%)	2.62 (1.48, 4.64)	0.001
RRT			
No	382 (95.0%)	1	
Yes	20 (5.0%)	1.85 (0.69, 4.97)	0.225
Alcohol consumption			
No	324 (80.6%)	1	
Yes	78 (19.4%)	1.32 (0.73, 2.40)	0.362
Diabetes mellitus			
No	341 (84.8%)	1	
Yes	61 (15.2%)	1.02 (0.51, 2.03)	0.954
Hypertension			
No	324 (80.6%)	1	
Yes	78 (19.40%)	0.89 (0.47, 1.68)	0.718
Cardiovascular disease			
No	381 (94.8%)	1	
Yes	21 (5.2%)	1.32 (0.47, 3.72)	0.601
Donation type			
DBD	170 (42.3%)	1	
DCD	232 (57.72%)	1.30 (0.78, 2.17)	0.310
Graft weight	1473 ± 362	1.00 (1.00, 1.00)	0.289
ABO compatibility			
ABO-i	46 (11.4%)	1	
ABO-c	356 (88.6%)	0.74 (0.36, 1.53)	0.413
RBC	4.0 (2.0–8.0)	1.02 (0.97, 1.07)	0.388
FFP	800 (630–1020)	1.00 (1.00, 1.00)	0.982
Auto-transfusion	250 (0–500)	1.00 (1.00, 1.00)	0.017
Intraoperative bleeding	1200 (800–2000)	1.00 (1.00, 1.00)	0.423
Intraoperative urine output	1050 (650–1600)	1.00 (1.00, 1.00)	0.053
Intraoperative RRT			
No	359 (89.3%)	1	
Yes	43 (10.7%)	1.96 (0.97, 3.96)	0.061
Post-transplant ammonia	49 (35–67)	1.01 (1.00, 1.01)	0.033
Post-transplant sedatives			
No	322 (80.1%)	1	
Yes	80 (19.9%)	1.39 (0.77, 2.50)	0.273

*p* < 0.05 was considered statistically significant. ABO-c, ABO compatible; ABO-i, ABO incompatible; BMI, body mass index; DBD, donation after brain death; DCD, donation after cardiac death; FFP, fresh frozen plasma; INR, international normalized ratio; RBC, red blood cell; RRT, renal replacement therapy.

**Table 3:** Relationship between total bilirubin and post-transplant delirium in different models.

Variable	Crude Model		Minimally Adjusted Model		Fully Adjusted Model	
	OR (95% CI)	*p* Value	OR (95% CI)	*p* Value	OR (95% CI)	*p* Value
Total bilirubin (per 1 mg/dL)	1.04 (1.02, 1.07)	<0.001	1.05 (1.02, 1.07)	<0.001	1.03 (0.99, 1.06)	0.104
Total bilirubin (tertiles)						
T1	Reference		Reference		Reference	
T2	2.15 (1.08, 4.31)	0.030	2.29 (1.14, 4.62)	0.020	2.02 (0.97, 4.22)	0.060
T3	3.80 (1.94, 7.44)	<0.001	4.20 (2.11, 8.38)	<0.001	2.46 (1.06, 5.69)	0.035
*p* for trend	<0.001		<0.001		0.035	

Crude model: did not adjust covariates. Minimally adjusted model: adjusted for age and gender. Fully adjusted model: adjusted for age, gender, creatinine, international normalized ratio, sodium, malignancy, encephalopathy, artificial liver support, auto-transfusion, intraoperative renal replacement therapy, and post-transplant ammonia.

**Table 4:** The results of two-piecewise regression model.

The Inflection Point of Total Bilirubin (Per 1 mg/dL)	OR	95% CI	*p* Value
≤20	1.05	1.01 to 1.11	0.026
>20	0.97	0.90 to 1.05	0.482

Effect: postoperative delirium. Cause: preoperative total bilirubin. Adjusted for age, gender, creatinine, international normalized ratio, sodium, malignancy, encephalopathy, artificial liver support, auto-transfusion, intraoperative renal replacement therapy, and post-transplant ammonia.

**Table 5:** Effect size of preoperative total bilirubin on postoperative delirium in prespecified and exploratory subgroups.

Characteristics	No. of Patients	OR (95% CI)	*p* for Interaction
Age, years			
≤55	255	1.03 (1.00, 1.07)	0.452
>55	147	1.01 (0.95, 1.08)	
Gender			
Male	339	1.03 (1.00, 1.07)	0.976
Female	63	1.02 (0.91, 1.16)	
BMI			
≤22	164	1.07 (1.01, 1.14)	0.006
>22	238	1.00 (0.96, 1.04)	
MELD			
≤22	271	1.06 (0.98, 1.15)	0.485
>22	131	1.00 (0.96, 1.05)	
Hepatitis B			
No	93	1.08 (1.00, 1.16)	0.388
Yes	309	1.02 (0.98, 1.06)	
Malignancy			
No	235	1.04 (0.99, 1.09)	0.201
Yes	167	1.00 (0.95, 1.05)	
Encephalopathy			
No	332	1.02 (0.99, 1.06)	0.115
Yes	70	1.00 (0.94, 1.07)	
Artificial liver support			
No	331	1.03 (1.00, 1.07)	0.036
Yes	71	0.99 (0.93, 1.05)	
Alcoholic consumption			
No	324	1.02 (0.99, 1.06)	0.870
Yes	78	1.06 (0.99, 1.15)	
Diabetes mellitus			
No	341	1.02 (0.99, 1.06)	0.959
Yes	61	1.12 (0.99, 1.27)	
Hypertension			
No	324	1.01 (0.98, 1.05)	0.375
Yes	78	1.10 (1.01, 1.19)	
RBC transfusion, u			
≤5	226	0.99 (0.94, 1.05)	0.234
>5	176	1.04 (0.99, 1.08)	
Auto-transfusion			
No	167	1.01 (0.96, 1.08)	0.358
Yes	235	1.03 (0.99, 1.07)	
Intraoperative bleeding, mL			
≤1000	183	1.01 (0.95, 1.07)	0.194
>1000	219	1.03 (0.99, 1.07)	
Intraoperative urine output			
≤1000	196	1.04 (1.00, 1.09)	0.892
>1000	206	1.01 (0.96, 1.07)	
Intraoperative RRT			
No	359	1.02 (0.99, 1.06)	0.589
Yes	43	1.07 (0.98, 1.17)	
Post-transplant ammonia			
≤49	209	1.02 (0.98, 1.06)	0.817
>49	193	1.05 (1.00, 1.10)	
Post-transplant sedatives			
No	322	1.03 (1.00, 1.07)	0.660
Yes	80	1.03 (0.96, 1.12)	

Note 1: Above model adjusted for age, gender, creatinine, international normalized ratio, sodium, malignancy, encephalopathy, artificial liver support, auto-transfusion, intraoperative renal replacement therapy, and post-transplant ammonia. Note 2: In each case, the model is not adjusted for the stratification variable. *p* < 0.05 was considered statistically significant. BMI, body mass index; MELD, model for end-stage liver disease; RBC, red blood cell; RRT, renal replacement therapy.

## Data Availability

The data that support the findings of this study are available from the corresponding author upon reasonable request.

## References

[1] Weiss N., Thabut D. (2019). Neurological Complications Occurring after Liver Transplantation: Role of Risk Factors, Hepatic Encephalopathy, and Acute (on Chronic) Brain Injury. Liver Transpl..

[2] Rengel K.F., Pandharipande P.P., Hughes C.G. (2018). Postoperative delirium. Presse Med..

[3] Zhou J., Xu X., Liang Y., Zhang X., Tu H., Chu H. (2021). Risk factors of postoperative delirium after liver transplantation: A systematic review and meta-analysis. Minerva Anestesiol..

[4] Brown C.H., Probert J., Healy R., Parish M., Nomura Y., Yamaguchi A., Tian J., Zehr K., Mandal K., Kamath V. (2018). Cognitive Decline after Delirium in Patients Undergoing Cardiac Surgery. Anesthesiology.

[5] Jin Z., Hu J., Ma D. (2020). Postoperative delirium: Perioperative assessment, risk reduction, and management. Br. J. Anaesth..

[6] Chen J., Wang H., He Z., Li T. (2020). Analysis of Risk Factors for Postoperative Delirium after Liver Transplantation. Neuropsychiatr. Dis. Treat..

[7] Austin C.A., O’Gorman T., Stern E., Emmett D., Stürmer T., Carson S., Busby-Whitehead J. (2019). Association between Postoperative Delirium and Long-term Cognitive Function after Major Nonemergent Surgery. JAMA Surg..

[8] Oliver N., Bohorquez H., Anders S., Freeman A., Fine K., Ahmed E., Bruce D.S., Carmody I.C., Cohen A.J., Seal J. (2017). Post-Liver Transplant Delirium Increases Mortality and Length of Stay. Ochsner J..

[9] Lingehall H.C., Smulter N.S., Lindahl E., Lindkvist M., Engström K.G., Gustafson Y.G., Olofsson B. (2017). Preoperative Cognitive Performance and Postoperative Delirium Are Independently Associated with Future Dementia in Older People Who Have Undergone Cardiac Surgery: A Longitudinal Cohort Study. Crit. Care Med..

[10] Bhattacharya B., Maung A., Barre K., Maerz L., Rodriguez-Davalos M.I., Schilsky M., Mulligan D.C., Davis K.A. (2017). Postoperative delirium is associated with increased intensive care unit and hospital length of stays after liver transplantation. J. Surg. Res..

[11] Beckmann S., Schubert M., Burkhalter H., Dutkowski P., De Geest S. (2017). Postoperative Delirium after Liver Transplantation is Associated with Increased Length of Stay and Lower Survival in a Prospective Cohort. Prog. Transplant..

[12] Inouye S.K., Westendorp R.G.J., Saczynski J.S. (2014). Delirium in elderly people. Lancet.

[13] Duning T., Ilting-Reuke K., Beckhuis M., Oswald D. (2021). Postoperative delirium-treatment and prevention. Curr. Opin. Anaesthesiol..

[14] Marcantonio E.R. (2017). Delirium in Hospitalized Older Adults. N. Engl. J. Med..

[15] Goldberg T.E., Chen C., Wang Y., Jung E., Swanson A., Ing C., Garcia P.S., Whittington R.A., Moitra V. (2020). Association of Delirium with Long-term Cognitive Decline: A Meta-analysis. JAMA Neurol..

[16] Mattison M.L.P. (2020). Delirium. Ann. Intern. Med..

[17] Park K.H., Son H.J., Choi Y.J., Park G.H., Lee Y.S., Park J.Y., Ri H.-S., Shim J.R. (2020). Liver Transplant Patients with High Preoperative Serum Bilirubin Levels Are at Increased Risk of Postoperative Delirium: A Retrospective Study. J. Clin. Med..

[18] Ri H.-S., Choi Y.J., Park J.Y., Jin S.J., Lee Y.S., Son J.-M., Yoon S.Z., Shin H.W., Choi B.H., Lee T.B. (2020). Elevation of Preoperative Ammonia Level Is Not Associated with the Incidence of Postoperative Delirium in Patients with Liver Transplantation: A Propensity Score Matching Analysis. Transplant. Proc..

[19] Hansen T.W.R., Wong R.J., Stevenson D.K. (2020). Molecular Physiology and Pathophysiology of Bilirubin Handling by the Blood, Liver, Intestine, and Brain in the Newborn. Physiol. Rev..

[20] Lescot T., Karvellas C., Chaudhury P., Tchervenkov J., Paraskevas S., Barkun J., Metrakos P., Goldberg P., Magder S. (2013). Postoperative delirium in the intensive care unit predicts worse outcomes in liver transplant recipients. Can. J. Gastroenterol..

[21] Wang S.-H., Wang J.-Y., Lin P.-Y., Lin K.-H., Ko C.-J., Hsieh C.-E., Lin H.-C., Chen Y.-L. (2014). Predisposing risk factors for delirium in living donor liver transplantation patients in intensive care units. PLoS ONE..

[22] Tavabie O., Colwill M., Adamson R., McPhail M., Bernal W., Jassem W., Prachialias A., Heneghan M., Aluvihare V.R., Agarwal K. (2020). A “real-world” analysis of risk factors for post liver transplant delirium and the effect on length of stay. Eur. J. Gastroenterol. Hepatol..

[23] Yoon J.S., Kim Y.R., Choi J.W., Ko J.S., Gwak M.S., Kim G.S. (2009). Risk factors of postoperative delirium following liver transplantation. Korean J. Anesthesiol..

[24] Watchko J.F., Tiribelli C. (2013). Bilirubin-induced neurologic damage—Mechanisms and management approaches. N. Engl. J. Med..

[25] Yueh M.-F., Chen S., Nguyen N., Tukey R.H. (2017). Developmental, Genetic, Dietary, and Xenobiotic Influences on Neonatal Hyperbilirubinemia. Mol. Pharmacol..

